# An advanced optical clearing protocol allows label-free detection of tissue necrosis *via* multiphoton microscopy in injured whole muscle

**DOI:** 10.7150/thno.51558

**Published:** 2021-01-01

**Authors:** Dominik Schneidereit, Anita Bröllochs, Paul Ritter, Lucas Kreiß, Zeinab Mokhtari, Andreas Beilhack, Gerhard Krönke, Jochen A Ackermann, Maria Faas, Anika Grüneboom, Sebastian Schürmann, Oliver Friedrich

**Affiliations:** 1 Institute of Medical Biotechnology, Friedrich-Alexander University Erlangen-Nürnberg, Paul-Gordan-Str. 3, 91052 Erlangen, Germany; 2 Graduate School in Advanced Optical Technologies (SAOT), Friedrich-Alexander University Erlangen-Nürnberg, Paul-Gordan-Str. 7, 91052 Erlangen, Germany; 3 Interdisciplinary Center for Clinical Research Laboratory for Experimental Stem Cell Transplantation, Department of Medicine II, Würzburg University Medical School, Zinklesweg 10, 97078 Würzburg, Germany; 4 Clinics of Internal Medicine III (Rheumatology and Immunology), University Hospitals Erlangen, Friedrich-Alexander University Erlangen-Nürnberg, Ulmenweg 18, 91054 Erlangen, Germany; 5 Muscle Research Centre Erlangen (MURCE), Henkestr. 91, 91052 Erlangen, Germany; 6 School of Medical Sciences (SOMS), University of New South (UNSW), Kensington Campus, High St, 2052 NSW, Sydney, Australia

**Keywords:** skeletal muscle, optical clearing, muscle injury, multiphoton imaging, light-sheet microscopy

## Abstract

**Rationale:** Structural remodeling or damage as a result of disease or injury is often not evenly distributed throughout a tissue but strongly depends on localization and extent of damaging stimuli. Skeletal muscle as a mechanically active organ can express signs of local or even systemic myopathic damage, necrosis, or repair. Conventionally, muscle biopsies (patients) or whole muscles (animal models) are mechanically sliced and stained to assess structural alterations histologically. Three-dimensional tissue information can be obtained by applying deep imaging modalities, *e.g.* multiphoton or light-sheet microscopy. Chemical clearing approaches reduce scattering, *e.g.* through matching refractive tissue indices, to overcome optical penetration depth limits in thick tissues.

**Methods:** Here, we optimized a range of different clearing protocols. We find aqueous solution-based protocols employing (20-80%) 2,2'-thiodiethanol (TDE) to be advantageous over organic solvents (dibenzyl ether, cinnamate) regarding the preservation of muscle morphology, ease-of-use, hazard level, and costs.

**Results:** Applying TDE clearing to a mouse model of local cardiotoxin (CTX)-induced muscle necrosis, a complete loss of myosin-II signals was observed in necrotic areas with little change in fibrous collagen or autofluorescence (AF) signals. The 3D aspect of myofiber integrity could be assessed, and muscle necrosis in whole muscle was quantified locally *via* the ratios of detected AF, forward- and backward-scattered Second Harmonic Generation (fSHG, bSHG) signals.

**Conclusion:** TDE optical clearing is a versatile tool to study muscle architecture in conjunction with label-free multiphoton imaging in 3D in injury/myopathy models and might also be useful in studying larger biofabricated constructs in regenerative medicine.

## Introduction

Current advances in our understanding of organ and tissue performance in health, disease, aging and tissue engineering are more and more dominated by the 'structure-determines-function' dogma, where the 3D-imprinted architecture of cellular cytoskeleton, extracellular matrix and multicellular arrangement provides a 'beyond signaling' frame for the mid- to the long-term performance of cells and tissues 1,2. Indeed, specific structural remodeling patterns on the cellular level have recently been experimentally documented to directly correlate with cellular performance, *e.g.* in single muscle cells, using a combined opto-biomechatronics approach 1. Unlike in single cells, structural assessment of tissue architecture in large volumetric organs or tissue components in 3D relies on (i) sectioning and (ii) contrast registry to be able to map a specific object pattern derived from optical contrast to a spatial position. For organs and tissues exceeding several orders of magnitude of cellular dimensions, the most common classical approach regarding (i) is mechanical sectioning of thin slices using microtomes to allow light penetration and reduce scattering, followed by (ii) enhancing contrast through externally applied labels and subsequent microscopy of sections to obtain 2D structural information 3. 3D reconstructions from serial slices are possible, however, they require extensive manual, labor-intensive and time-consuming processing and image data reconstruction that can also be prone to artifacts in image registration and thus, structural data loss or misinterpretations 4,5. The most limiting factor for optical penetration into tissues is given by light-matter interactions that reduce the incident (or emitted) light intensity or redirect the light path within the tissue by the processes of absorption and scattering, respectively. Although absorption of light by specific labels introduced to the sample is desired, tissue eventually may also contain intrinsic fluorophores (*e.g.* melatonin, hemoglobin, myoglobin, etc.) that also unspecifically absorb incoming light. Although scattering primarily changes the direction of light without energy loss, this may also cause a point source of light from within tissue to broaden its extension, while destructive interferences from distant scatterers contribute markedly to the translucency of tissues 6. Thus, conventionally, aiming for tissue sections thick enough to maintain structural integrity within, but thin enough to keep scattering to a minimum, is a trade-off that is often encountered. Tissue penetration is also a wavelength-dependent function, with near-infrared light penetrating deeper into tissue than visible light 7. For visible light confocal laser-scanning imaging, scattering in muscle tissue can already become so marked, that depth limits correspond to the diameter of one single cell layer 8. This limit can be extended by a factor of two to three in turbid tissue media 8,9, using infrared two-photon excitation imaging. Although this can be beneficial for *in vivo* imaging 10, the penetration depths will still be limited to a fraction of the tissue thickness. The limited penetration means that regional or deeper changes to tissue architecture, *e.g.* remodeling after injury, may remain undetected below the surface. Going from optical coherence tomography (OCT) to photo-acoustics imaging or micro-computer tomography does increase tissue penetration, however, at the cost of resolution. OCT, for instance, has an optical penetration depth of 2 mm but a lateral resolution of 10 µm 11. Thus, for high-resolution tissue structural analyses, it is still 'either the blade' or reduction of light scattering.

Attenuation of scattering through chemical clearing (aka optical clearing) has become an indispensable tissue processing technique to increase the transparency of whole organs or even small organisms (*e.g.* embryos) by minimizing refractive index mismatches at interfaces. This alignment of refractive indices throughout the sample usually requires an elevation of the refractive index (RI) of the tissue fluids (extracellular/intracellular) by exchanging water for high RI solutions and/or dehydration, as well as by delipidation, depending on the technique used. In general, optical clearing approaches can be grouped into solvent-based and aqueous solution-based chemical treatments. The approaches may be further sub-grouped into delipidation and dehydration/hyperhydration followed by RI matching, or hydrogel embedding followed by delipidation and RI matching 12-14. In principle, one can choose from a plethora of chemical reagents, bioprocess recipes and incubation times as well as commercial optical clearing compounds. However, in general, clearing conditions must be optimized according to the target organ specifications and imaging goals. Very detailed quantitative studies have been provided for solid and hollow organs 13,14. As a rule of thumb, most expectations towards an ideal optical clearing protocol are set out to fulfil the following claims: (i) highest transparency possible with maximum optical penetration depths, (ii) compatibility with external/genetically encoded fluorescent dyes, (iii) preservation of 3D architecture by the processing, (iv) short processing times, (v) low to no toxicity of the involved clearing agents and (vi) low cost 15. For instance, while solvent-based clearing agents (*e.g.* 3DISCO, uDISCO) 4,16, detergent/urea clearing (CUBIC) and hydrogel embedded clearing (*e.g.* PACT) are usually associated with deeper penetration depths following clearing, this comes at the cost of more pronounced tissue geometry changes across several soft tissues and organs when compared to aqueous clearing agents 13,14. Solvent-based clearing methods also show generally worse fluorescent staining preservation than aqueous clearing agents, but recent advanced techniques, like PEGASOS, prove to maintain even protein fluorescence well 17. However, organic solvents are still generally more toxic and can even compromise imaging equipment 12. Thus, aqueous clearing agents have more recently experienced growing attention to developing recipes for organ clearing that is less hazardous, easy to perform and of low cost 12,14,18. Although clearing capacity may be lower as compared to solvent-based clearing agents, immersion in some (but not all) aqueous solutions with high RI has been considered advantageous in terms of procedure handling, clearing time, preservation of sample structure and fluorescent proteins 6,12. The absence of detergents preserves the lipid content in those approaches, but aqueous solution-based clearing can be combined with detergents (*e.g.* SDS, Triton X-100 or urea) to optionally remove lipids or denature proteins with success 6. The intracellular water content of cells is replaced by the high RI clearing solution *via* osmotically driven diffusion in immersion clearing. Hereby the RI of the sample bulk is adapted toward RI values observed in proteins and lipids (~1.43) 12,19. Among those low-cost reagents having been widely promoted for simple immersion clearing are polyols, like sucrose, fructose, glycerol and 2,2'-thiodiethanol (TDE) 6,20. Some of the immersion clearing substances, like saccharides or glycerol, exhibit very low toxicity and have, therefore, been successfully applied for optical clearing *in vivo* or optical clearing of explanted functional tissue 21,22. However, the currently published methods do not appear powerful enough to attempt imaging of whole functional muscle. In addition to the mentioned simple reagents, there are also more complex ready-to-use commercial clearing solutions available (*e.g.* FocusClear^TM^) 3. TDE, in particular, has the advantage of being water-soluble and of low viscosity. Furthermore, its RI can be tuned by dilution in water 6,23. TDE has been used successfully in fast clearing (~30 min) of fixed mouse brain 24, mouse hippocampus 25, human placenta 26 or tissue spheroids 12. At high concentrations, TDE may compromise fluorescence of several fluorophores 23, but lower concentrations and/or fractional increases in concentration incubation were described to largely preserve fluorescence, i.e. of genetically-encoded probes 24. Nevertheless, since most of the deep tissue imaging to assess parenchymal architecture involves multiphoton imaging, in particular *Second Harmonic Generation* (SHG), the non-linear frequency-doubling effect of biological molecules, such as fibrous collagen and myosin-II, can be label-free assessed in conjunction with optical clearing 21,26-28.

Skeletal muscle is the archetype of an organ where both SHG-susceptible proteins, myosin-II and collagen, constitute to a large extent to the intracellular cyto- and extracellular matrix-architecture, respectively. SHG has been widely used in recent years to study the 3D aspects of muscle tissue in health 21,29-31, during aging 32-34, and disease 32-36. For instance, in primary genetic myopathies with a chronic degenerative clinical course (i.e. muscular dystrophy, myofibrillar myopathy), we have developed a so-called 'quantitative morphometry' approach to identify specific myofibrillar and sarcomeric myosin-II and extracellular fibrosis (collagen-I) remodeling patterns and applied to a disease or aging entity 1,32,33,37. Although most of these studies have focussed on SHG-changes in single muscle fibers, they are likewise applicable to larger tissue sections 38 and even whole intact muscle 39. However, only to depths of a maximum of ~400 µm in native tissue (less so for forward scattered SHG detection ~150 µm) 39.

In order to overcome the depth limitations of SHG imaging in native tissue, we combined multiphoton molecular fingerprinting for myofibrillar myosin-II and extracellular matrix fibrous collagen SHG and cellular autofluorescence with immersion-based optical clearing in the present study. The aqueous TDE and solvent-based ethyl cinnamate (ECi) and dibenzyl ether (DBE) agents were chosen for our investigation as they fulfil the criteria of being (i) widely used, (ii) non-proprietary, (iii) of low toxicity, (iv) simple in composition and (v) application, (vi) quick, and they promise to maintain (vii) sample fluorescence and (viii) sample structure well. Another goal was to facilitate studies of skeletal muscle damage and remodeling (in particular in models of localized injury), and to better understand differential affections of muscle disease, aging or repair in inflammatory or degenerative myopathies. For this, we tested and optimized several optical clearing protocols for their applicability in whole murine skeletal limb muscle. As an injury model, we chose local cardiotoxin injection that is known to induce complete muscle necrosis in the vicinity of the injection site, sparing satellite cells as the source for subsequent regeneration 40. Although different models of muscle injury and regeneration have been employed in the past, so far damage and regeneration have been mostly assessed based on stem cell counts and histochemistry analysis for fiber diameters and fibrosis in tissue sections, although one study also provides 3D-morphometry of GFP-labelled vessels 41. However, even though depth analysis up to 150 µm for vessel architecture could be retrieved, the myofiber structural architecture could not be captured. Thus, our approach provides new venues for deep muscle cellular architecture analysis and quantitative details related to myosin sarcomeric signal distribution in muscle necrosis with destroyed myofibrillar cytoarchitecture.

## Methods

### Animal handling and muscle preparation

All animals were handled in accordance with the German Animal Welfare Act ('Tierschutzgesetz'), and the investigations were approved by the Government Office for Animal Care and Use (Regierungspräsidium Unterfranken; 55.2 DMS-2532-2-97). Muscle samples were obtained from C57BL/6N mice of between 23 and 26 weeks old animals. 50 µl cardiotoxin (CTX, Latoxan, Portes-lès-Valence, France) solution (10 µM in PBS) was injected into the *musculus tibialis anterior* (TA) of one hind limb while the respective muscle of the contralateral side was injected with 50 µl PBS three days before muscle extraction. Injections were performed with a 30-gauge needle under anesthesia. Muscle isolation was performed on the severed hind limbs in a Sylgard^®^ (Sylgard 184, DOWSIL, Midland, USA)-coated culture dish in Ringer's solution. The extracted muscles were transferred to individual 1.5 ml centrifuge tubes that contained 1 ml Phosphate-buffered saline solution (PBS, PH 7.4) with 3.7% Paraformaldehyde (PFA) for fixation and storage.

### Physical sectioning and H&E staining

Muscles were fixed in 4% (w/v) paraformaldehyde overnight, stored in 70% ethanol for at least 12 h, and embedded in paraffin before sectioning. 2 µm Sections were cut longitudinally and stained with hematoxylin and eosin.

### Applied chemical clearing protocols

**Figure 1:**
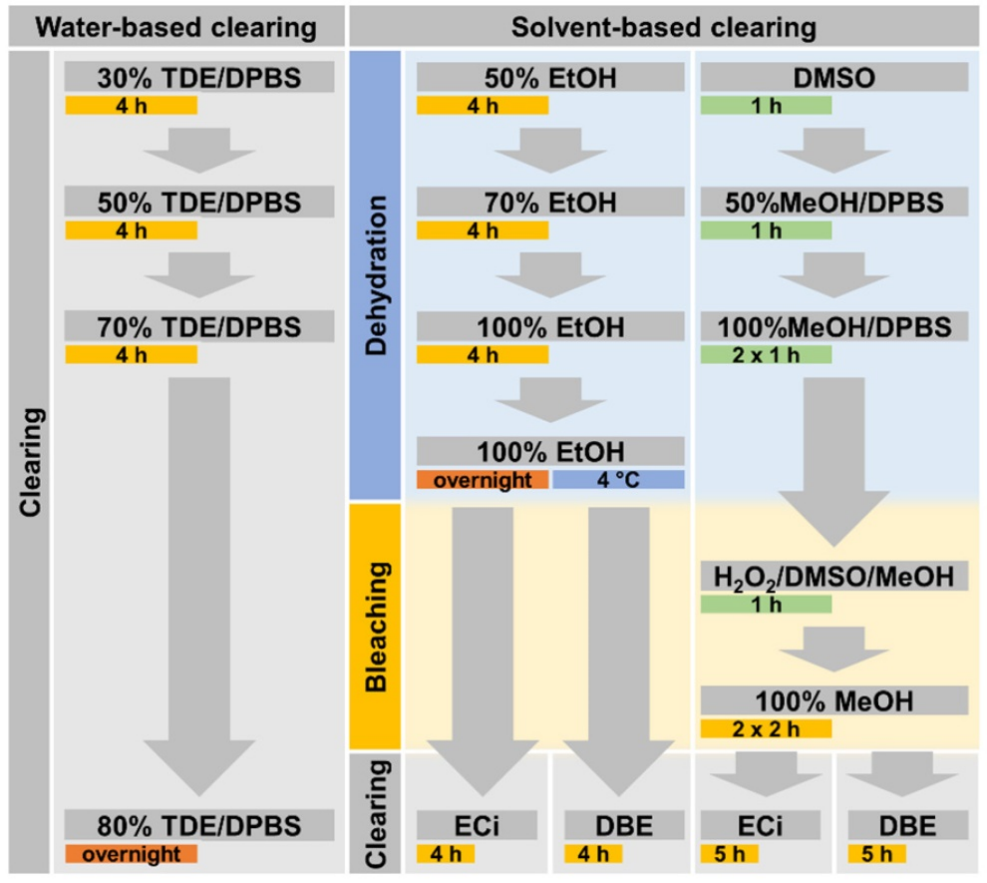
** Flowchart of applied clearing procedures.** Incubation of each processing step is performed for the specified time during slight sample shaking and at room temperature, if not specified otherwise. The TDE-based clearing procedure is applied in aqueous solution while DBE- as well as ECi-based clearing both require an initial dehydration procedure.

The clearing procedure was initiated after fixing the muscle samples for at least 2 h at room temperature. All clearing procedure steps were performed in 2 ml centrifuge tubes that were filled with 1.5 ml of liquid unless otherwise specified. The clearing procedures are visualized as a flowchart in Figure **1**.

### TDE clearing

The solution in the centrifuge tubes was replaced four times with Dulbecco's Phosphate-Buffered Saline (DPBS) solution containing increasing amounts of 2,2'-thiodiethanol (TDE) and incubated for 4 h at room temperature (RT under gentle shaking (30 rpm) after each exchange. The applied TDE concentrations were 30%, 50%, 70% and 80% (v/v). After the final solution exchange, the sample was incubated overnight at 4°C with gentle shaking (30 rpm) and following that, stored at 4 °C until imaging. The procedure was adapted from Costantini *et al.* (2015) 25 and Aoyagi *et al.* (2015) 24.

### Ethanol-based dehydration

The fixing solution in the centrifuge tubes was replaced with ultra-pure water containing 30% (v/v) of ethanol and incubated for 2 h at RT under gentle shaking (30 rpm). The solution was exchanged four more times, increasing ethanol concentration each step to 50%, 70% 99.8% and again 99.8% (v/v). The sample was incubated for 4 h at RT after each solution exchange and then incubated overnight at RT (all under gentle shaking). Samples were stored at RT until clearing with either DBE or ECi. The dehydration procedure was adapted from published procedures 20,42,43.

### Methanol-based dehydration and bleaching

The fixing solution was replaced five times with ultra-pure water containing increasing concentrations of methanol (MeOH) followed by a 1 h incubation at RT and gentle shaking. The applied MeOH concentrations were 30%, 50%, 80%, 99.9% and 99.9% (v/v). After the final incubation period, the solution was exchanged for a bleaching mix consisting of MeOH with 5% (v/v) of hydrogen peroxide. The sample was incubated overnight (4 °C, gentle shaking) and thereafter, the solution was exchanged to 99.9% MeOH and incubated for 4 h (RT, gentle shaking) before initiating either DBE or ECi clearing. The procedure was adapted from Klingberg *et al.* (2017) 20 and Renier *et al.* (2014) 42.

### Dibenzyl ether (DBE) clearing

After either methanol or ethanol-based dehydration, the sample solution was replaced with DBE and incubated for 6 h (RT, gentle shaking). The sample was stored at RT until imaging.

### Ethyl cinnamate (ECi) clearing

After either methanol- or ethanol-based dehydration, the sample solution was replaced with ECi and incubated for 6 h (RT, gentle shaking). Following the incubation, the sample was stored at RT until imaging. As the melting point of ECi is 8 °C, ECi immersed samples were kept at RT at all times.

### Imaging setup

*Second Harmonic Generation* imaging (SHG) was performed using a multiphoton microscope (TriMScope II, LaVision BioTec, Bielefeld, Germany). Microscope specifications, as well as image analysis capability of ultrastructural architecture, are described in Schneidereit *et al.* (2018) 1. SHG and autofluorescence (AF) signals were excited using a mode-locked ps-pulsed Ti:Sa laser (Chameleon Vision II, Coherent, Santa Clara, USA) tuned to 810 nm wavelength. The mean laser output power during imaging ranged between 200 and 350 mW. The mean laser power on the sample in the objective focal plane was 40-80 mW. The laser power was adapted according to the sample requirements to allow distinguishing sample features with sufficient signal-to-noise ratio. The transmitted fSHG, as well as the backscattered bSHG signal, were detected using a 405/20 nm single bandpass filter (Chroma Technology group, Acal BFi Germany GmbH, Germany) and an ultrasensitive, non-descanned transmission photomultiplier tube (PMT) (H 7422-40 LV 5 M, Hamamatsu Photonics, Japan). The samples' two-photon AF was recorded using a 525/50 nm (Chroma Technology group, Acal BFi Germany GmbH, Germany) single bandpass filter and a non-descanned PMT. Both bSHG and AF were detected in the backward direction through the excitation objective. The signal was separated from the excitation light *via* a 700 nm long pass dichroic mirror, and the signal light was further split using a 495 nm long pass dichroic mirror. A 25x HC FLUOTAR L (Leica Microsystems GmbH, Germany) objective was used on the excitation side of the setup, and the light was collected in transmission direction through a U-AAC condenser lens (OLYMPUS, Japan). The recorded images had a voxel size from 0.4 x 0.4 x 2 µm to 0.8 x 0.8 x 10 µm. Light-sheet fluorescence microscopy (LSFM) was performed on a custom-built LSFM set-up as described 44. The applied voxel size was 1x1x2 µm using an HCX APO L20x/0.95 IMM objective (*Leica*, Wetzlar, Germany) in conjunction with a 525/50 nm emission filter and a Neo 5.5 camera (*Andor*, Belfast, UK). The excitation laser wavelength was 488 nm.

### Absorbance spectra measurements

Sample absorbance was determined using the NanoDrop unit of a spectrophotometer (Jasco V630 SAH-76, JASCO Applied Sciences, Halifax, Canada) and placing the muscle sample in the beam path of the 1 mm wide defined gap between the glass coverslips of the measurement cell. The muscle samples were immersed in their respective clearing fluid during absorbance measurements. The spectra were acquired from 200 - 800 nm with data points acquired each 1 nm. The optical bandwidth at each measured point was 1.5 nm. However, due to strong scattering in the short-wavelength UV range, measurements were evaluated only from 400 nm.

### Image- and statistical analysis

The mean sarcomere length was determined in the 2D Fourier transform of each image slice of acquired 3D stacks using *Fiji* 45. The image intensity of the channels of image slices in 3D stacks was obtained using *Fiji*. The intensity ratios were calculated for each image slice, using the mean pixel intensity value of each image channel. Statistical data analysis was performed using *SigmaPlot* (Built 13.0.0.83).

## Results

### Optimization of optical clearing protocols for deep, label-free, two-photon (2p) imaging and quantitative morphometry of whole mouse muscles

**Figure 2:**
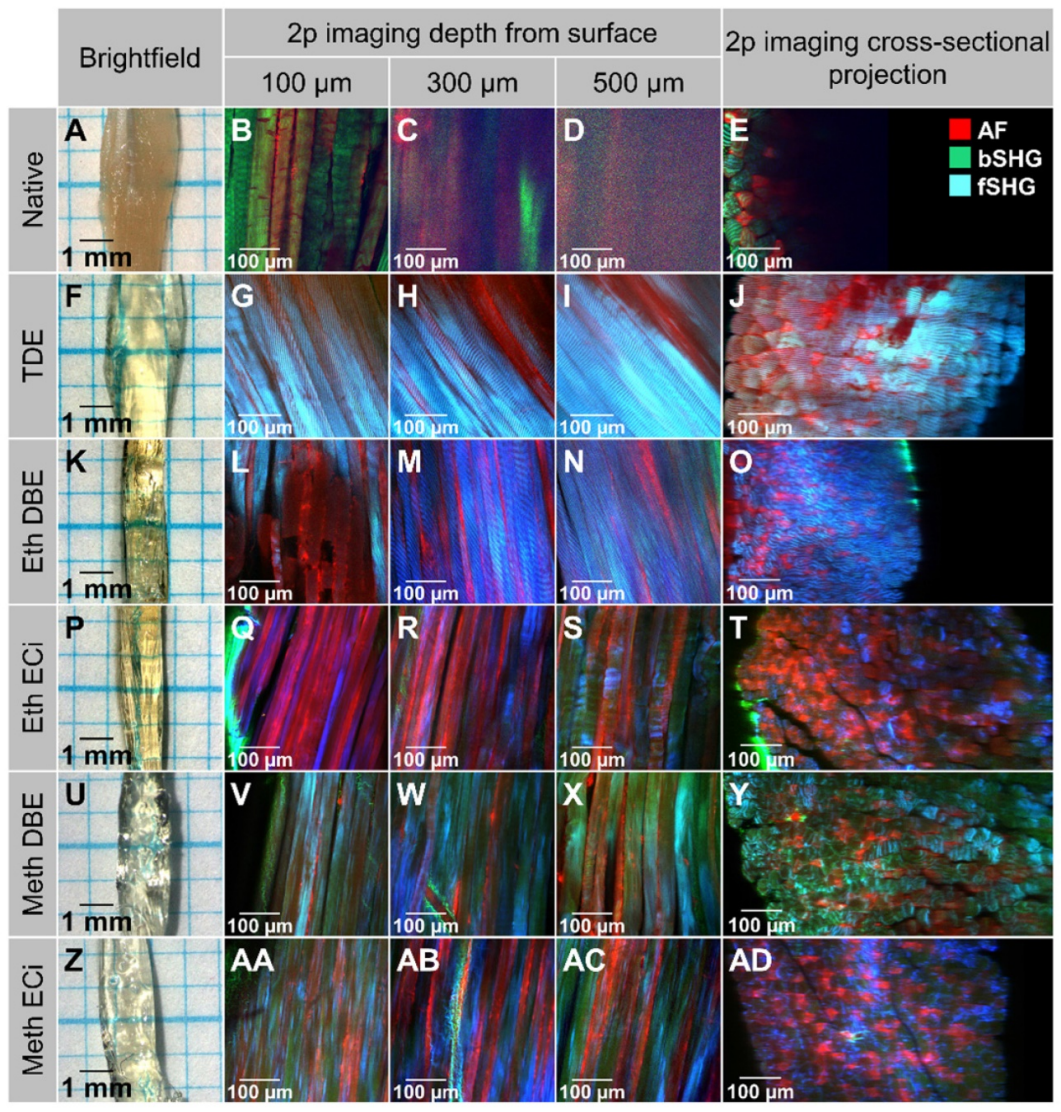
** Comparison of different optical clearing protocols on bright-field and 2p-microscopy imaging in whole mouse EDL muscle.** The bright-field images (left lane) of all clearing techniques show increased transparency. Solvent-based approaches induce tissue shrinkage due to dehydration steps. Multiphoton image slices, taken at different sample depths, as well as reconstructed vertical cross-sectional projection images (XZ), are acquired without physical sectioning or staining of the muscle, and illustrate the effects of clearing protocols on penetration depth and signal retention. The sample autofluorescence is recorded at 525 nm and is shown in red. *Second Harmonic Generation* (SHG) signal is recorded at 405 nm in backward scattering (green), predominantly representing fibrous collagen ECM matrix architecture, and forwards scattering (blue), representing myosin-II myofibrillar sarcomere architecture, direction. A high-resolution version of the image is available in the supplements as S04.

**Figure 3:**
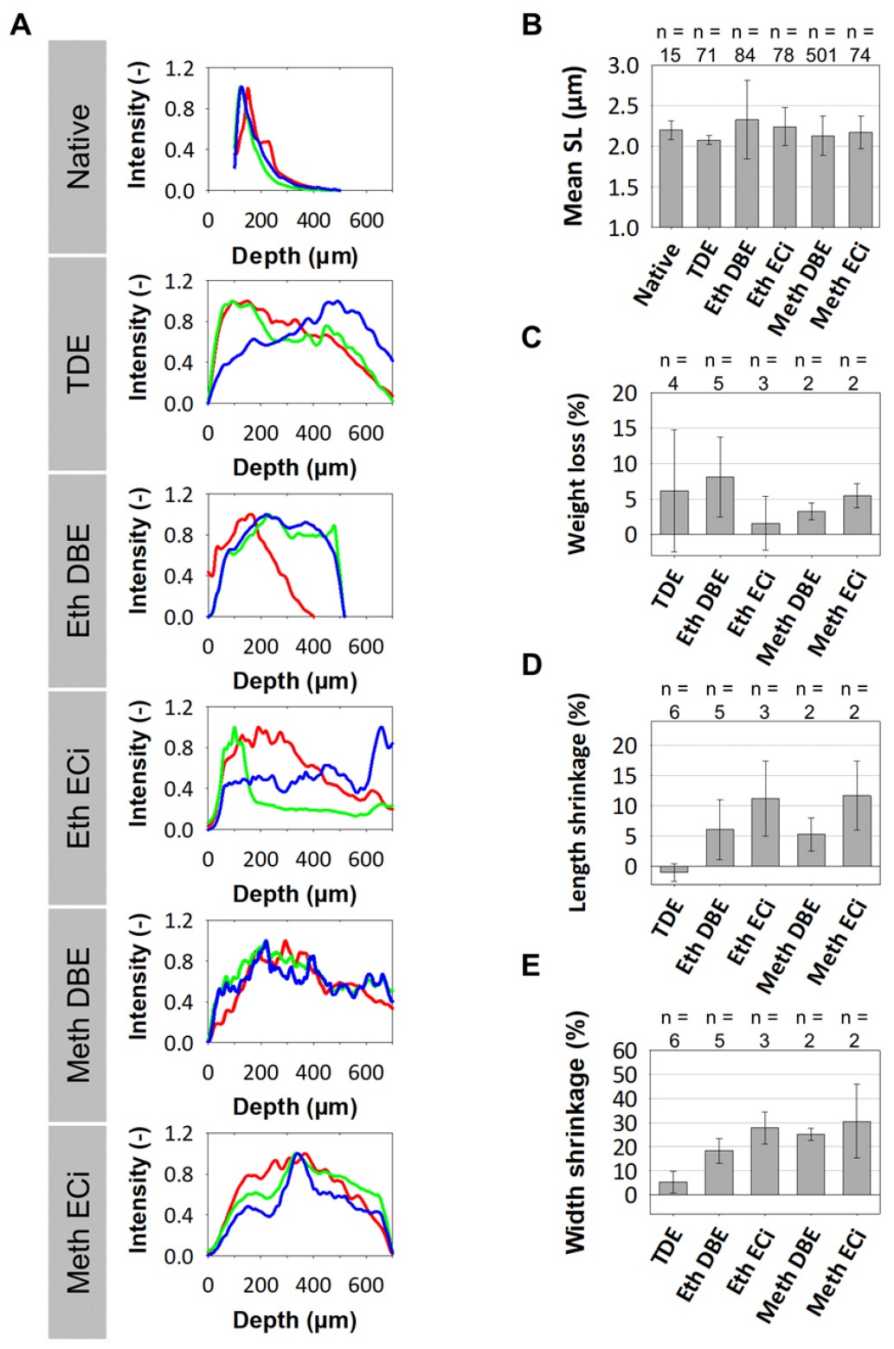
** Morphometric data of optical clearing protocols affecting signal penetration depths, sarcomere length and muscle macroscopic size and weight. A,** For the muscle volumes of **Figure 2**, the depth profile of the label-free generated 2p-AF (red), fSHG (blue) and bSHG (green) are shown. While signal intensity declines exponentially in native muscle, intensity is best preserved in depth in TDE and DBE-treated muscles. **B,** average sarcomere lengths analyzed through the whole XYZ volumes by automated morphometry in the EDL muscles show no significant difference to the native muscle (ANOVA Tuckey). The number n represents the number of evaluated single images. Comparing pooled EDL and *soleus* muscles pre- and post-treatment, only TDE-treatment was capable in preserving macroscopic tissue parameters, as weight **(C)**, length **(D)** and organ width **(E)**. The number n represents the number of individual evaluated muscles. All error bars from **(B)-(E)** represent the standard deviation.

**Figure 4:**
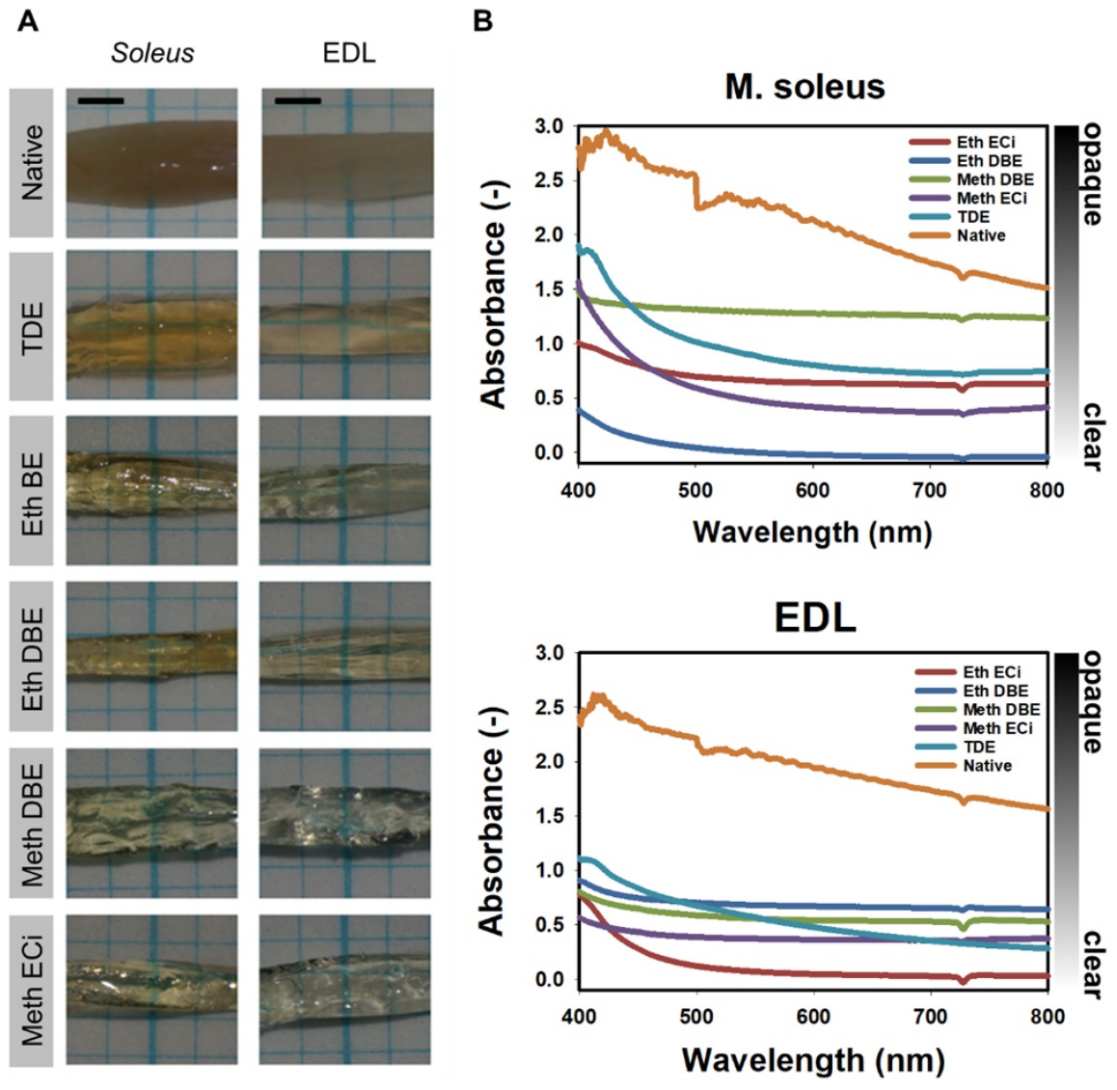
** Transparent appearance and spectral absorbance of whole mouse *soleus* and EDL muscles. A,** Brightfield images of whole mouse EDL and *soleus* muscles following the optical clearing protocol indicated. **B,** spectral absorbance curves for the muscles shown in **(A)**.

Promising protocols from literature applied to soft tissues were identified, and two solvent-based and one aqueous solution-based clearing agent identified as a starting point for further optimization of whole muscle optical clearing. For the latter, 2,2'-thiodiethanol (TDE) was chosen and for the former, dibenzyl ether (DBE) and ethyl cinnamate (ECi), respectively. **Figure 1** visualizes the flowchart of the three procedures. While the TDE-clearing is relatively simple, the solvent-based protocols require pre-treatment of the samples through dehydration and bleaching. Either method takes up to 2 days for completion **(Table S2)**. Native mouse *extensor digitorum longus* (EDL) muscles without any treatment showed the regular reddish opaque appearance resulting from apparent light scattering in the tissue. In those, label-free 2p-imaging was only able to deliver informative structural image data from depths up to ~200 µm, from whereon scattering detrimentally reduced signal resolution in deeper layers **(Figure 2, Figure 3A)**. All tested protocols resulted in a noticeable clearing of the whole muscle which seemed more effective in the order Meth DBE/ECi > TDE > Eth DBE/ECi (**Figure 2**, brightfield). Label-free 2p-imaging allowed to separate non-linear signals according to cellular autofluorescence (AF), forward-scattered SHG (fSHG) and back-scattered SHG (bSHG). All clearing protocols resulted in a marked extension of imaging depth, with clear images well exceeding 600 µm **(Figure 2)**. However, not all clearing procedures seemed to preserve the intrinsic cyto- and matrix-architecture as judged in particular from the fSHG signal patterns, originating from myosin-II composed myofibrils. Although all protocols allowed to image through the whole muscle following clearing, as indicated by the section panel in **Figure 2**, TDE and Eth-ECi reproduced myosin-II originating fSHG best, while the signal was less pronounced throughout the samples for the other protocols. This is unlikely to be caused by a solvent-induced reduction in sarcomere length (SL) as fSHG signal scales with SL 29. **Figure 3B** shows that sarcomere lengths of the samples shown in **Figure 2** were not statistically different from the native (uncleared) sample. The penetration depth for the AF, fSHG and bSHG signal intensities fall off exponentially in the native sample with a depth constant of about 200 µm. In contrast, the penetration depth remained high throughout the whole sample depth in the optically cleared samples. Best results were noted in the TDE and DBE samples, followed by the ECi samples (note that the muscle shown for the Eth DBE sample was relatively small with a thickness around 0.5 mm, reflected by the sharp signal drop at 500 µm where the muscle ended). Mouse *soleus* whole muscles were also subjected to the respective clearing protocols as an example of a slow-twitch muscle. Although *soleus* muscle is in general somewhat larger than the EDL, optical clearing by pure diffusion resulted in similar transparency results **(Figure 4A)**. Importantly, when analyzing whole muscle morphological parameters, TDE turned out to be the most preserving treatment in both muscles with almost no weight loss or shrinkage, unlike the other protocols that resulted in even up to 60% weight loss and substantial shrinkage due to dehydration **(Figure 3 C‑E)**. A comparison of the absorbance spectra of both muscles following the given optical clearing procedures **(Figure 4B)** reveals that TDE clearing was associated with an at least two-fold increase in transmittance (decrease in absorbance) compared to the native specimen although other clearing protocols, in particular DBE and ECi clearing, even further improved optical transparency across the whole visible spectrum.

**Figure 5:**
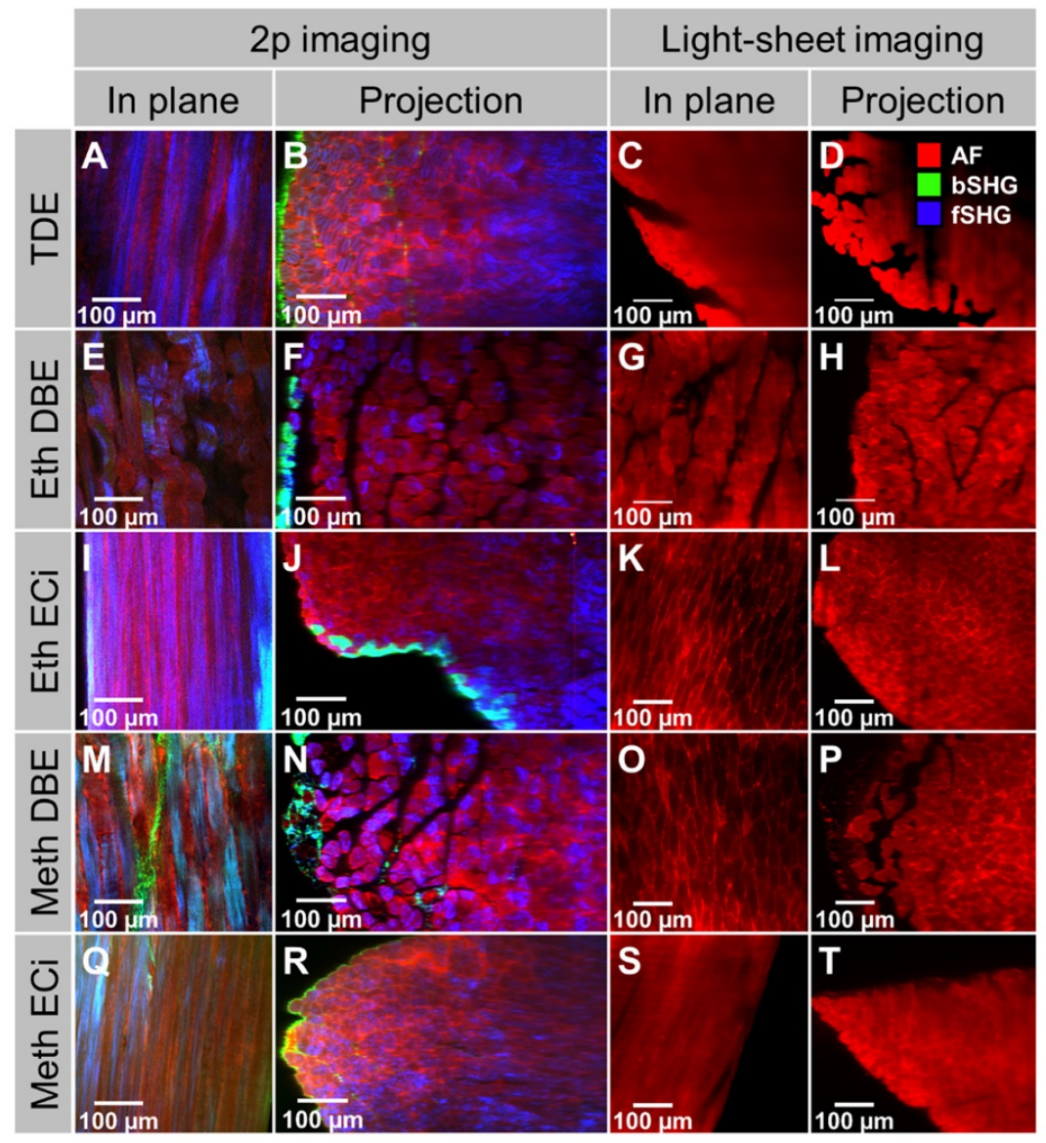
** Comparison of the same optically cleared whole muscles (* soleus*) under label-free 2p- and 1p light-sheet microscopy shown as images slices in the focal plane and cross-sectional projections.** For all treatments shown, the 2p-scanning excitation provided not only a much better penetration depth, i.e. through the whole muscle, over the 1p-light-sheet excitation, but also shows the advantage of multi-spectral signal collection from both AF and SHG (f/bSHG). While the structural architecture is also reproduced in the light-sheet images, the molecular identity of signals is missing. Thus, the light-sheet images only contain the tissues' AF signals. A high-resolution version of the figure is available in the supplements as S05.

Light-sheet, or single-plane illumination (SPIM), microscopy is an imaging modality that employs a 90° visualization of the 1p-induced Gaussian excitation beam. It is often performed in conjunction with optical clearing since the 1p-Gaussian beam allows deep imaging of thick tissues, whole organs, and even small embryos when clearing is applied. As 1p-SPIM is not a point scanning procedure, it is considerably faster than 2p-laser scanning microscopy. However, since in the current study of analyzing optically cleared whole muscles, imaging speed was not an issue, we sought to compare both modalities using the very same samples. The absence of artificially introduced external or genetically encoded fluorescent dyes in the native optically cleared muscle only allows for tracking autofluorescence from the sample in 1p-SPIM, while in 2p-imaging the molecular signature of the signal origin is preserved. **Figure 5** demonstrates this in whole mouse *soleus* muscles that were subjected to 2p-scanning microscopy (AF + SHG) and 1p-light-sheet imaging, respectively, following the various optical clearing procedures. Two things can be noted here: (i) 2p-excitation still allowed deeper penetration depths as compared to 1p-SPIM, and (ii) the structural integrated signal architecture of the 2p-images is reproduced in the 1p-light-sheet images, albeit with no further molecular signal identity. Thus, in optically cleared whole organs where structural assessment prevails (no live-cell imaging), two-photon imaging is preferable over 1p-SPIM for label-free assessment. From these assessments of optical clearing performance, a qualitative classification for whole mouse muscle is presented in **Table 1**.

### 3D structural assessment of whole muscle architecture in a model of skeletal muscle injury and necrosis (label-free 3D-2p-quantitative morphometry)

**Figure 6:**
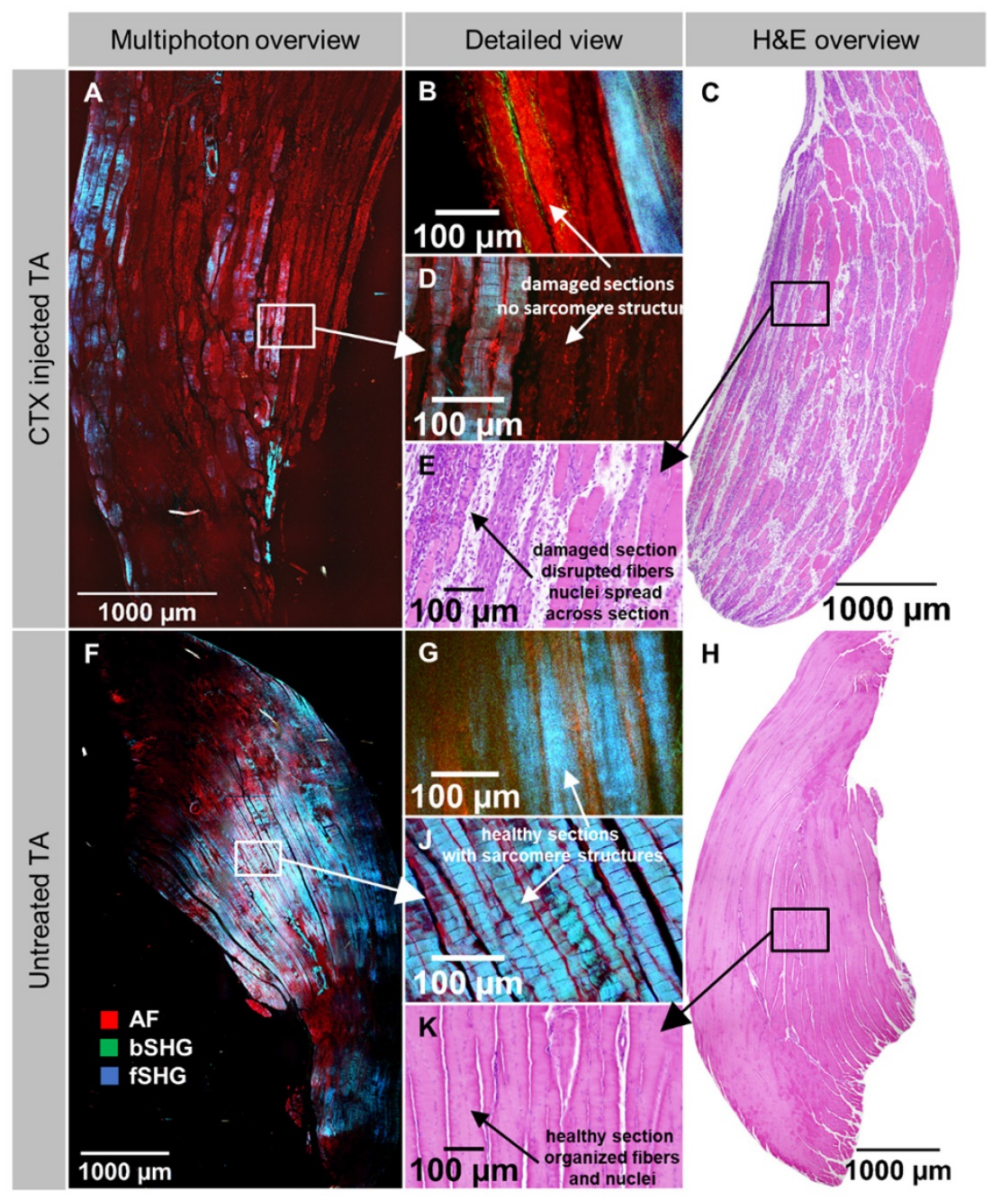
** Label-free 2p structural aspect of *tibialis ant.* muscle following *in vivo* cardiotoxin (CTX)-injection to induce localized muscle necrosis.** Whole muscles were dissected at day 3 following CTX-injection into living mice and subjected to label-free 2p-microscopy following either fixation and mechanical slicing for H&E histology or TDE optical clearing of whole unsliced muscles. Large field-of-view stitched images from mechanically sliced sections of the muscle from a CTX-treated (A) and sham-treated (F) leg (different animals) as well as higher-resolved images around the injection site (D), or from within the normal parenchyma (J) are shown. Note that the necrotic area is characterized by a sharp loss of fSHG myosin-II structural signal with unspecific cellular AF remaining and also widened intercellular space that is also backed up by the H&E histology images showing necrotic areas with loss of cellular structure and starting inflammatory infiltration (C, E). In the sham-treated muscle, intercellular space is equally distributed. A, D, F, J are unstained 2p images from an unstained histology slice. B, G are optical slices from within a 2p-imaged unstained and optically cleared whole muscle XYZ volume. The wavy patterns of fibers seen in the mechanically sliced sections (D, J) but not from within XYZ volumes (B, G) most likely result from artefacts following embedding and mechanical slicing.

From the previous section on clearing protocols optimization, we consider TDE-clearing to be the most suitable for whole mouse muscle clearing, preserving tissue architecture and providing an excellent tissue penetration for label-free 2p-morphometry. This could be applied to a plethora of various disease models and tissue damage or repair studies, for which we chose a very standardized local muscle necrosis and regeneration model using myotoxic cardiotoxin injection into the muscle of live mice. As described in the Methods, whole muscles were excised three days post-injection where necrosis is expected to be well developed, subjected to TDE-optical clearing, and 2p-imaged. As a comparison with conventional H&E staining, also unstained, mechanically thin-sliced fixed muscle sections were subjected to label-free 2p-imaging. **Figure 6** shows representative large field-of-view (FOV) stitched images from such an unstained mechanically sliced section of *tibialis ant.* (TA) muscle from a CTX-injected **(A)** and a sham-injected leg **(F)** alongside with respective magnified views **(D, J)**. Intriguingly, around the injection site where necrosis is most predominant, a highly selective loss of myofibrillar SHG signal (fSHG) can be detected with a sharp demarcation line even between two myofibers. Intact myofibers still show the non-linear fSHG signal while in necrotic fibers, only unspecific AF signal remains. Areas in the magnified sections **(Figure 6F, Figure 6J)** showed 'wavy' SHG patterns in both conditions that most probably reflect artifacts resulting from the embedding and mechanical slicing procedure and are not inherent to the CTX-treatment. The direct comparison with optical 2p-slices obtained from whole optically cleared muscles supports this observation **(Figure 6B, G)** where no such 'wavy' artifact patterns were seen. Nevertheless, the fSHG signal, representing the myofibrillar myosin-II, is present throughout both the optically scanned and mechanically sliced samples. The label-free multiphoton architecture is also backed up by additional H&E-histology on CTX-injected and untreated TA muscles. The former showed large streaks of lacunae running through the muscle (**Figure 6C**), indicative of necrotic areas with also inflammatory infiltrates **(Figure 6E)** while the pattern was regular and evenly structured with no infiltrates in the sham-control **(Figure 6H, K)**. Note that the H&E images were from muscles from different animals than the 2p-images due to different processing.

**Figure 7:**
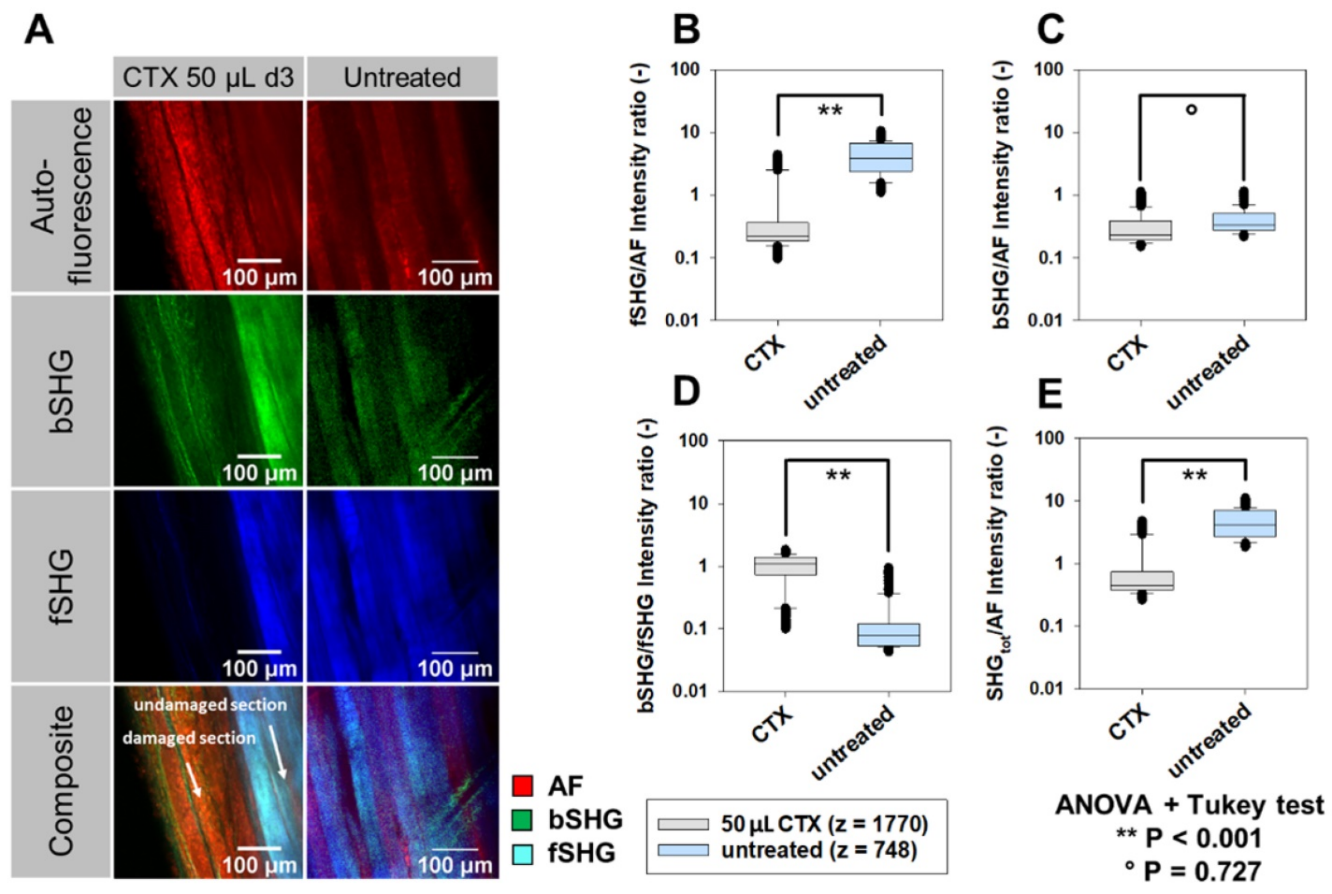
** 3D label-free 2p AF and SHG signal intensity analysis in whole XYZ volumes of necrotic *tibialis ant****.* muscle three days following cardiotoxin (CTX)-injection. Whole muscles, dissected at day 3 following CTX-injection into living mice, were subjected to label-free 2p-microscopy (AF, SHG) following TDE-clearing. A, AF, bSHG, fSHG and composite overlay images obtained from within the TDE-cleared CTX-treated and sham-treated (untreated) muscle showing the sharp demarcation line of the necrotic area which is best visualized in the composite image where the relative decline in fSHG over AF signal colorizes the necrotic area in orange over the lighter blue undamaged section. SHG signals were ratioed against AF signals (B, C, E) as well as for bSHG:fSHG (D) to account for differences in AF signal across imaging depths and between muscles. A significant specific loss of myosin-II fSHG signal intensity is apparent in the CTX-treated muscle (B, D, E) while the predominantly collagen bearing bSHG signal is similar in necrotic compared to untreated muscle (C). The number z refers to the actual number of image slices per data set obtained from three complete XYZ volumes of each one muscle pair.

In order to analyze signal intensities and structural morphology more quantitatively in necrotic muscle, 2p-signal intensities **(Figure 7)** were computed. **Figure 7A** shows representative images taken from an XYZ volume image through a whole TDE-cleared CTX-treated (images are shown at depth 260 µm) and sham-treated muscle (depth: 370 µm) that were subjected to label-free AF, fSHG and bSHG imaging. A sharp demarcation line, delineating the necrotic area three days following CTX-treatment within the tissue, can most impressively be seen in the fSHG images at the depth where fibers with intact fSHG signal pattern neighbor with fibers void of fSHG signal, indicative of lost myofibrillar sarcomere structure. Due to the relatively preserved AF signal intensity in both areas, this colorizes the necrotic area as orange over the light blue undamaged area in the composite image. For a more quantitative analysis and to account for AF signal intensity fluctuations across the depth and between muscle areas and muscles imaged, the respective SHG signals and their total integrated intensity were normalized to AF for each image, as well as bSHG:fSHG intensities were ratioed and are presented in **Figure 7B‑E**. Statistical analyses from almost 2,500 image slices of several volume images through different areas of CTX-treated and untreated TDE-cleared muscles show a highly significant loss of fSHG signal intensity, both when normalized to AF **(Figure 7B)** and bSHG **(Figure 7D)**. Unlike the loss in myofibrillar sarcomeric myosin-II signal, the predominantly fibrous collagen bearing bSHG signal intensity was not altered in necrotic tissue **(Figure 7C)**. The same is also evident from the total SHG signal over AF **(Figure 7E)**, thus demonstrating the power of label-free two-photon imaging to detect necrosis in muscle tissue at depth. Videos showing representative reconstructed 3D image stacks of both CTX-treated (**S1**) and untreated muscle (**S2**) are provided in the supplements.

## Discussion

The need for minimally invasive, ideally label-free, deep imaging of tissues and organs to 3D-reconstruct tissue morphology to study disease patterns, aging or maturation of bioartificial, engineered tissues, has driven a plethora of chemical approaches in the last years to reduce light scattering as the primary limiting optical constraint in dense turbid tissue 13,14. As such, good optical transparency and ease-of-use, reproducibility, short treatment times, as well as non-toxicity, are all aspects to consider for the feasibility of a specific procedure. Most previous research has focused on whole small animals or their solid and hollow organs, in particular brain, lung, heart 13,24, some even used human heart samples 15,46, small spheroids 12 or developing embryos 47. Although a very detailed recent study quantitatively compared a large selection of whole mouse organs for the efficiency of optical clearing and penetration depths in confocal microscopy, mostly solvent-based or more complex aqueous-based procedures have been employed 14. Simple, fast and structure-preserving chemical aqueous clearing agents, in particular, 2,2'-thiodiethanol have been applied to whole mouse brains 24,25, allowing to resolve fine neuronal details to depths of up to 2 mm in two-photon excited fluorescence microscopy 24. In skeletal muscle, to our knowledge, no studies have been performed using the advantageous properties of TDE, while studies both employing solvent-based and other aqueous protocols have been carried out, for instance, to explain mechanisms of optical clearing in muscle tissue (see discussion below). The lack of existing investigations prompted us to compare several established muscle clearing protocols with TDE-clearing and employ label-free two-photon SHG and AF-imaging and quantitative analysis of signal patterns not only to optically cleared muscle 'per se' but also to apply it to demonstrate its capabilities in a clinically relevant model of local muscle injury and necrosis. In particular for the latter, our study represents the first one to provide optically-obtained molecular fingerprint information for necrotic muscle at depth. Our main findings here are: (i) TDE clearing provides superb f/bSHG and AF-SNR intensities for depths of at least 600 µm and exceeding **(Figure 2, Figure 3)**, (ii) TDE has best muscle morphological preservation properties with regard to shrinkage **(Figure 3)**, (iii) TDE-2p imaging reproduced microscopic tissue patterns seen with 1p light-sheet imaging but providing label-free molecular fingerprint signals **(Figure 5)**, and (iv) the TDE-2p imaging approach was able to reproduce necrosis hallmarks seen in H&E histology slices from CTX-injected muscle but with molecular identity originating from the signal channels and much finer detail **(Figure 6)** that (vi) allowed specific quantitative necrosis assessment from 2p-signals in 3D **(Figure 7)**.

### Optical penetration depth and structural preservation in cleared muscle

In the study of Xu *et al.* (2019) 14, a range of optical clearing procedures (3DISCO, uDISCO, SeeDB, FRUIT, ScaleS, CUBIC, PACT) was used to optically clear several mouse organs (hollow and solid), among them *gastrocnemius* muscle. In the kidney, they found confocal microscopy imaging depths up to 1,300 µm by solvent-based 3DISCO and uDISCO while aqueous solutions performed worse (up to 300 µm, note: TDE was not among them). In another study using EtOH-ECi cleared kidneys, an imaging depth of 2,500 µm was yet achieved, applying confocal laser scanning microscopy 20. For the hollow organ intestine, the difference 'solvent vs. aqueous' was less obvious, as the necessary sample penetration depth was only around 300 µm 14. Although for muscle, the solvent-based clearing was concluded to be preferred over aqueous procedures, no data on imaging depth was provided 14. However, one also needs to keep in mind the mostly toxic nature of many solvent-based agents 6. As one example of simple immersion in aqueous glycerol-containing solutions, Plotnikov *et al.* (2006) 21 used SHG imaging and found a 2.5-fold increase in achievable imaging depth using 50% glycerol solutions in healthy mouse *gastrocnemius* and *quadriceps femoris* muscle to about 210 µm (85 µm in uncleared muscle): optical clearing was fast and almost complete after 2 h and not further improved by 24 h treatments 21. However, it came along with significant swelling of muscle fibers within the tissue 21,48. By increasing glycerol concentrations to 75% 48 or 100% 21, representing a RI of 1.43 and 1.45, respectively, imaging depth was not substantially improved and worse after 24 h of clearing which prompted the authors to conclude that the final optically cleared result was not the sole result of refractive index matching. In fact, glycerol has been known to break down membranes, which is associated with a loss of proteins from the myoplasm and, thus, a reduction of the inner filter effect of proteins as the predominant mechanism in optically cleared unfixed tissue. Prior fixation, however, prevented the loss of proteins and concomitant reduction in the inner filter effect 21. In our case, we did not attempt to use unfixed muscle samples for optical clearing, mostly for logistic reasons. However, transferring those conclusions to our setting in fixed muscles, clearing through TDE may most likely have occurred through RI match (RI between 1.4 and 1.5, concentration-dependent from 30% to 97%, Richardson & Lichtman 2015). In ethylene glycol (EG)-induced clearing of thin muscle samples of 0.5 mm, initial dehydration within the first minute of the optical clearing was noted in collimated optical transmittance recordings as the predominant mechanism in aqueous EG-clearing that is then replaced by RI matching taking over 49. The same group described a roughly 65% improvement in tissue spectral transmittance in rat muscle following 20 min of diffusion-clearing with ethanol-glycerol-*aqua dest.* mixtures 50.

Apart from increasing imaging depth, optical clearing of rat *quadriceps* muscle in 50% glycerol (n ~ 1.47) also preserved the angular polarization dependence of SHG signal of myosin-II through depths of 100 µm (with some depolarization evident at depths of 180 µm). In contrast, polarization was already highly randomized following 2-3 collision lengths (~50 µm) in the uncleared muscle 51. Although SHG intensity varies with the angle of input polarization to the myofibrillar myosin axis 29, which may strongly affect projected penetration depths deduced from the forward scattered SHG signals, we took care to orient muscle specimen within an angular variation of between 0° and a maximum of ~45° with respect to the input polarization, which would only result in a variability of SHG signal intensity of 15% in the worst case (in the lower ~100 µm depth range) or even none at all in the best case (at ~180 µm depth or deeper, 51). SHG polarization dependence was not a focus in the present study but will be addressed in subsequent TDE clearing studies. As one example of hydrogel-embedded muscle fixation and clearing using the CLARITY protocol, Milgroom & Ralston (2016) 52 confirmed preservation of myofibrillar muscle myosin-II and fibrous collagen SHG signals in mouse EDL muscles as well as two-photon fluorescence, however, only to depths of 100 µm which already improved penetration depths about 2.5-fold. Detailed depth limits were, unfortunately, not addressed 52. In a study comparing Clear^T2^ (PEG-formamide, 12 h clearing), ScaleA2 (urea/glycerol/Triton-X-100, 2 weeks clearing) and 3DISCO (dehydration, dibenzyl ether, 1 h clearing) clearing of mouse muscle (*quadriceps, gastrocnemius*) and bioartificial muscle constructs (fibrin or collagen hydrogels containing HUVEC cells) on the penetration depth of GFP signals assessed by confocal fluorescence imaging, ScaleA2, and 3DISCO significantly improved penetration depth from about ~150 µm to between 300 µm and 400 µm compared to uncleared samples, while Clear^T2^ seemed to be ineffective 53. Clear^T2^ and ScaleA2 mostly preserved the size of the muscle specimen 53, while 3DISCO resulted in significant shrinkage of the tissue 14. On the other hand, 3DISCO and Clear^T2^ represented relatively fast clearing options for muscle within less than one day as compared to ScaleA2 that required two weeks 53. Of note, in another study on ScaleA2 clearing applied to whole mouse brains, ScaleA2 was associated with marked swelling of the brain during five days of clearing 54. Lastly, solvent-based effects of protein denaturation have to be considered in addition to dehydration to impact on SHG signal intensities or even change the non-linear optical properties of biological molecules. For instance, ethanol dehydration applied to HeLa cells was demonstrated to induce cellular DNA transition from B to A form associated with a change from invisible to visible under SHG microscopy 55.

Apart from our imaging approach, we also provide spectral intensity analyses of whole muscle absorbance following our different optical clearing approaches. Of those, TDE produced a roughly two times drop in absorbance across the 400 - 800 nm spectral range, both for EDL and *soleus* muscle. Although other clearing agents produced even better transparency (i.e. DBE, ECi), the structural preservation and optical penetration depth for infrared excitation light (810 nm) and SHG emission light (405 nm) for TDE outperformed other clearing approaches. In the literature, only very limited data on spectral absorbance/transmittance properties are available. In a study focusing on immersion clearing of rat abdominal wall muscle with an ethanol-glycerol-*aqua dest* composition (1:1:2), a swift increase in transmittance within 30 s was observed 50. Their spectra also showed a transmittance increase with wavelength, which corresponds to the decrease in absorbance seen in our spectra **(Figure 4)**.

Major advantages are given through the structure preservation that is well documented for immersion approaches over solvent-based clearing techniques 6 when comparing our TDE-clearing approach to the above-mentioned studies. Although other aqueous clearing agents have been shown to induce complete clearing after shorter incubation times (hours-days) as compared to TDE (days-weeks) 6, the TDE clearing not only convinces through its high optical penetration through whole muscle tissue but also ease-of-use and non-toxicity.

### 3D assessment of muscle structure following optical clearing in muscle injury and disease models

Although systemic muscle disorders are expected to affect all skeletal muscle in the body, *e.g.* in genetic or chronic degenerative myopathies, the severity of structural derangement of muscle architecture, both from the extracellular matrix side and the actomyosin protein lattice, may vary substantially. For instance, Duchenne Muscular Dystrophy (DMD) is a genetic myopathy where the complete absence of the protein dystrophin induces recurrent degeneration and regeneration cycles that are associated with tissue architecture remodeling, loss of function and progressive weakness. Different muscles may be differentially affected, but the structural disorder of myofibrillar orientation 1,35, as well as fibrosis 56, are commonly seen. Optical clearing has been employed in methyl salicylate cleared EDL muscles from dystrophic *mdx* mice following sequential ethanol dehydration to visualize fiber branching within the organ using confocal microscopy 57. Although optical penetration depths, clearing agent concentration or incubation times were not given in that study, extensive branching of dystrophic fibers was also detected within the tissue column, which previously was mostly addressed in isolated single fibers only. In the field of muscle injury and repair, we are not aware of any study that has employed optical clearing alongside with label-free *Second Harmonic Generation* imaging to define sensitive optical parameters to delineate intact from injured (necrotic) tissue. In a distantly related study, Zeng *et al.* (2014) 58 used SHG and THG imaging in zebrafish *in vivo* to monitor tissue injury during immune reaction induced by bacterial infection. Their wounded regions were also void of SHG signal (Figure 3 in 58). However, their focus was on the assessment of neutrophils within the tissue. On a morphological level, our label-free multiphoton assessment well-reproduced features seen in conventional histology in CTX-injury models where between day 1 and day 4, necrosis was found to be most pronounced 41. However, unlike 2D cross-sections of necrotic and injured muscle, our multiphoton imaging allows the detailed 3D assessment and the delineation of exact borders between necrotic and uninjured tissue on the cellular level exploiting the myosin-II forward scattered SHG signals. Those seem to be very sensitive in reflecting the necrotic remodeling process on the intracellular level of damaged muscle fibers. In contrast, the extracellular matrix, reflected predominantly by collagen backscattered SHG signal, is not affected at all in the necrotic process. This may certainly change during the following repair and regeneration phase where also extracellular fibrosis or scar tissue formation may be involved but is clearly beyond the 3 day limit of the current study. However, this interesting aspect will be addressed in a follow-up study extending the observation range to one month.

A very novel outcome of our label-free imaging approach is the introduction of sensitive morphometry parameters to differentiate necrotic from healthy tissue quickly. As such, we focused on a ratio analysis of the AF, bSHG, and fSHG signals that quantitatively reflect the behavior mentioned above of loss of intracellular myosin-II signal at a maintained fibrous collagen signal in muscle necrosis. Such parameters can be quickly extracted from the image channels and plotted as ratios using automated image processing tools developed during our study. As such, this strategy could be useful for future augmentation of digital pathology employing SHG imaging in patient muscle biopsies or prospectively, even using minimal-invasive multiphoton endomicroscopy following further refinement and miniaturization of our recently introduced system 59.

## Conclusions

Optical clearing using an immersion-based thiodiethanol formulation has proven effective in achieving high optical transparency with enhanced penetration depths for two-photon imaging while preserving structural architecture and avoiding tissue swelling or shrinkage. The clearing process can be reduced to pure diffusion-penetration over a time duration of one to two days with good results. Since TDE is non-toxic and a cheap compound, this also argues in its favor. Our 3D quantitative morphometry approach in cardiotoxin-injured muscle reliably reproduces necrosis aspects seen in conventional histopathology but providing a label-free and specific molecular contrast inherent to intrinsic signal generators (myosin-II, fibrous collagen) throughout even a whole small EDL muscle (Figure S03). Necrotic areas are void of the fSHG signal, while bSHG remains unaffected by necrosis. In comparison to immunostaining or fluorescent protein expression, using the SHG signals forgoes the problematic need to deliver an antibody stain into the tissue 60 or genetically modify the investigated organism. With the application of our procedure to patient muscle biopsies, future studies will help to expand the optical analysis in 3D to include automated image processing and analysis with the help of machine learning approaches to contribute to digital pathology. Another interesting field of application in future experiments is the area of thick biofabricated tissue constructs in regenerative medicine and tissue engineering.

## Supplementary Material

Supplementary movie 1.

Supplementary movie 2.

Supplemental image 1.

Supplemental high-quality figure 2.

Supplemental high-quality figure 5.

## Figures and Tables

**Table 1:** Qualitative summary of clearing properties of the tested optical clearing protocols. bSHG: back-scattered SHG. fSHG: forward-scattered SHG. PFA: paraformaldehyde fixation.

	un-cleared	TDE	Eth DBE	Eth ECi	Meth DBE	Meth ECi
Clearing effect	no	yes	yes	yes	yes	yes
Auto-fluorescence	weak	good	weak	good	good	good
bSHG	↑↑↑	↑↑	↑	↑	↑	↑
fSHG	↑	↑↑	↑↑	↑↑	↑↑↑	↑↑
Shrinkage	some	no	yes	yes	yes	yes
Imaging depth	poor	good	fair	great	great	good
