# Supplementary material for: An advanced optical clearing protocol allows label-free detection of tissue necrosis via multiphoton microscopy in injured whole muscle
Source: Theranostics. 2021 Jan 1;11(6):2876–91. doi: 10.7150/thno.51558 (PMC7806485; doi:10.7150/thno.51558)
Supplement: Supplementary file 3 — Supplemental image 1. [file thnov11p2876s3.pdf]

Supplemental image 1

3D reconstruction of a 3D mosaic of an untreated whole EDL muscle. AF (525/50 nm) is shown in red, bSHG (405/20 nm) shown in green, and fSHG (405/20 nm) shown in blue. The Collagen-rich tendon exhibits strong bSHG and low AF. The muscle itself shows strong Myosin fSHG with patches of increased and reduced AF.

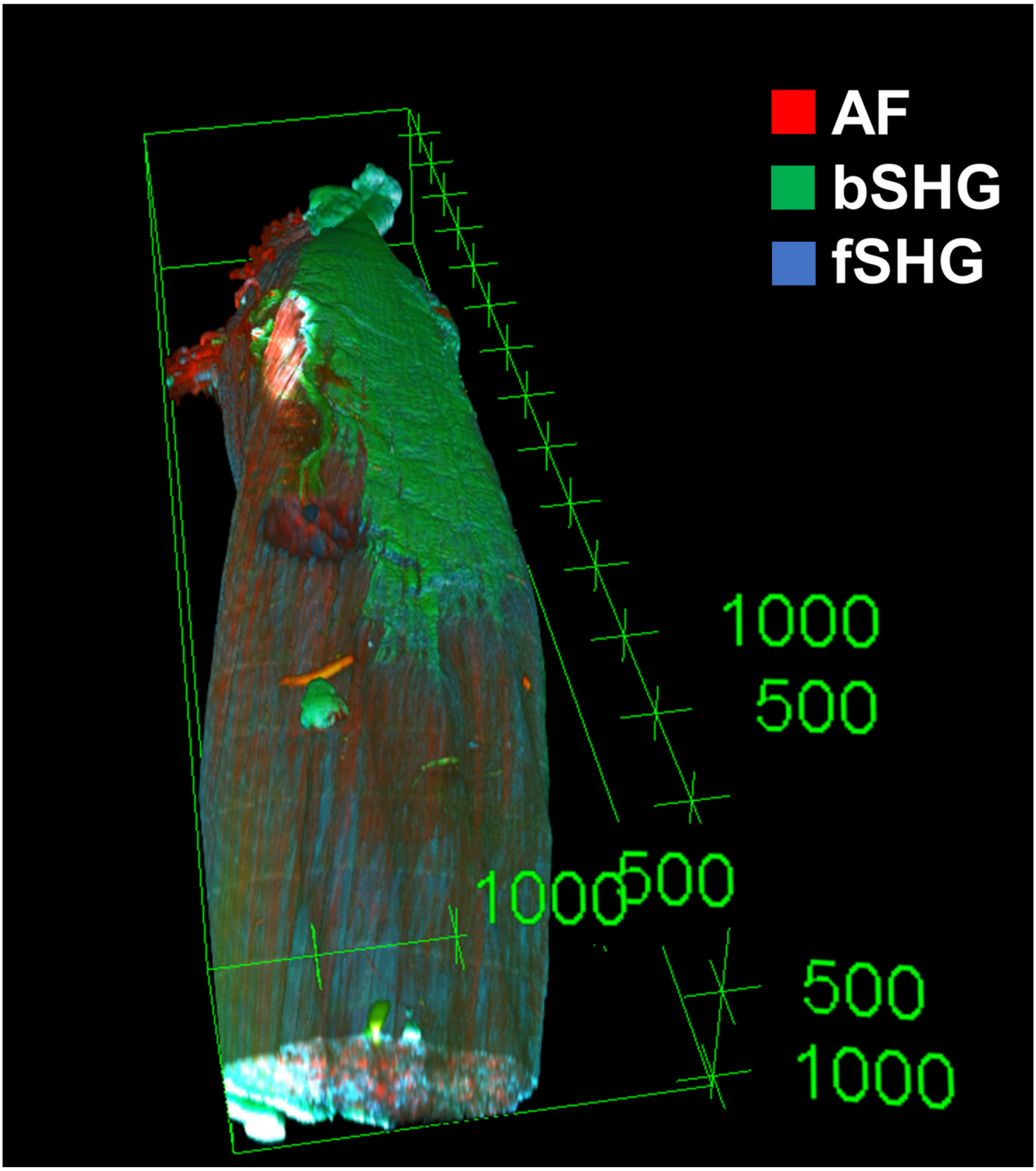

Supplemental high-quality figure 2

Fig 2 is provided as an uncompressed version to allow for a detailed comparison of each sub-images microstructures.

Supplemental high-quality figure 5

Fig 5 is also provided as an uncompressed version to allow for a detailed comparison of each sub-images microstructures.

Supplemental movie 1

3D reconstruction of an imaged TA muscle segment that has been treated with CTX. AF (525/50 nm) is shown in red, bSHG (405/20 nm) shown in green, and fSHG (405/20 nm) shown in blue. A subvolume of the muscle that is devoid of bSHG from sarcomere patterns is showing mostly AF signal. When the AF channel is faded out, muscle fibers that retain an intact sarcomeric pattern are revealed more clearly.

Supplemental movie 2

3D reconstruction and fly-through of an imaged TA muscle segment of the untreated control group. AF (525/50 nm) is shown in red, bSHG (405/20 nm) shown in green, and fSHG (405/20 nm) shown in blue. Muscle fibers with sarcomeric fSHG signal are visible throughout the whole segment, and sarcomeric patterns are discernible when zoomed in.
