# Supplementary figures and images for: An advanced optical clearing protocol allows label-free detection of tissue necrosis via multiphoton microscopy in injured whole muscle
Source: Theranostics. 2021 Jan 1;11(6):2876–91. doi: 10.7150/thno.51558 (PMC7806485; doi:10.7150/thno.51558)

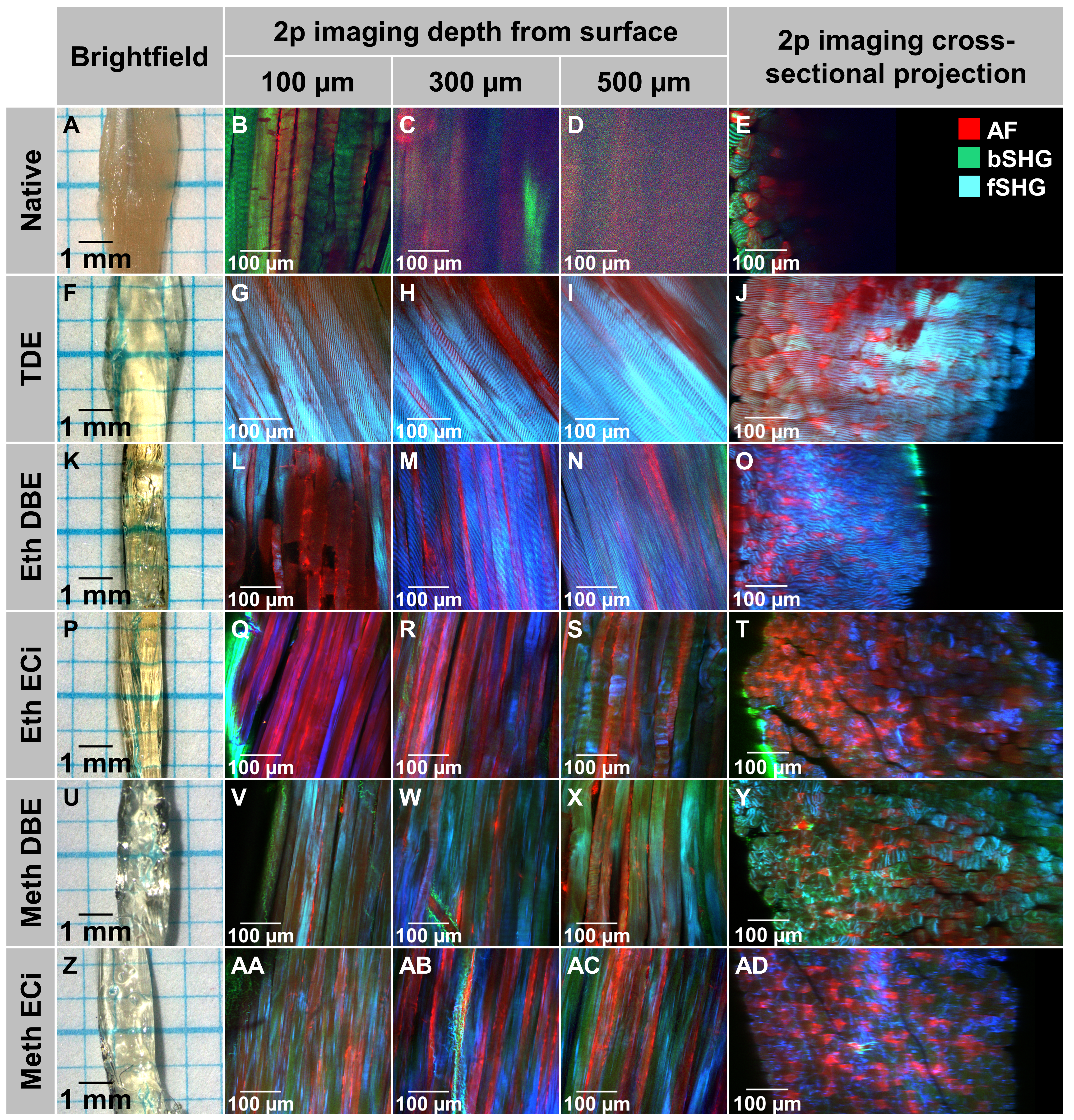

Supplement: Supplementary file 4 — Supplemental high-quality figure 2. [file thnov11p2876s4.png]

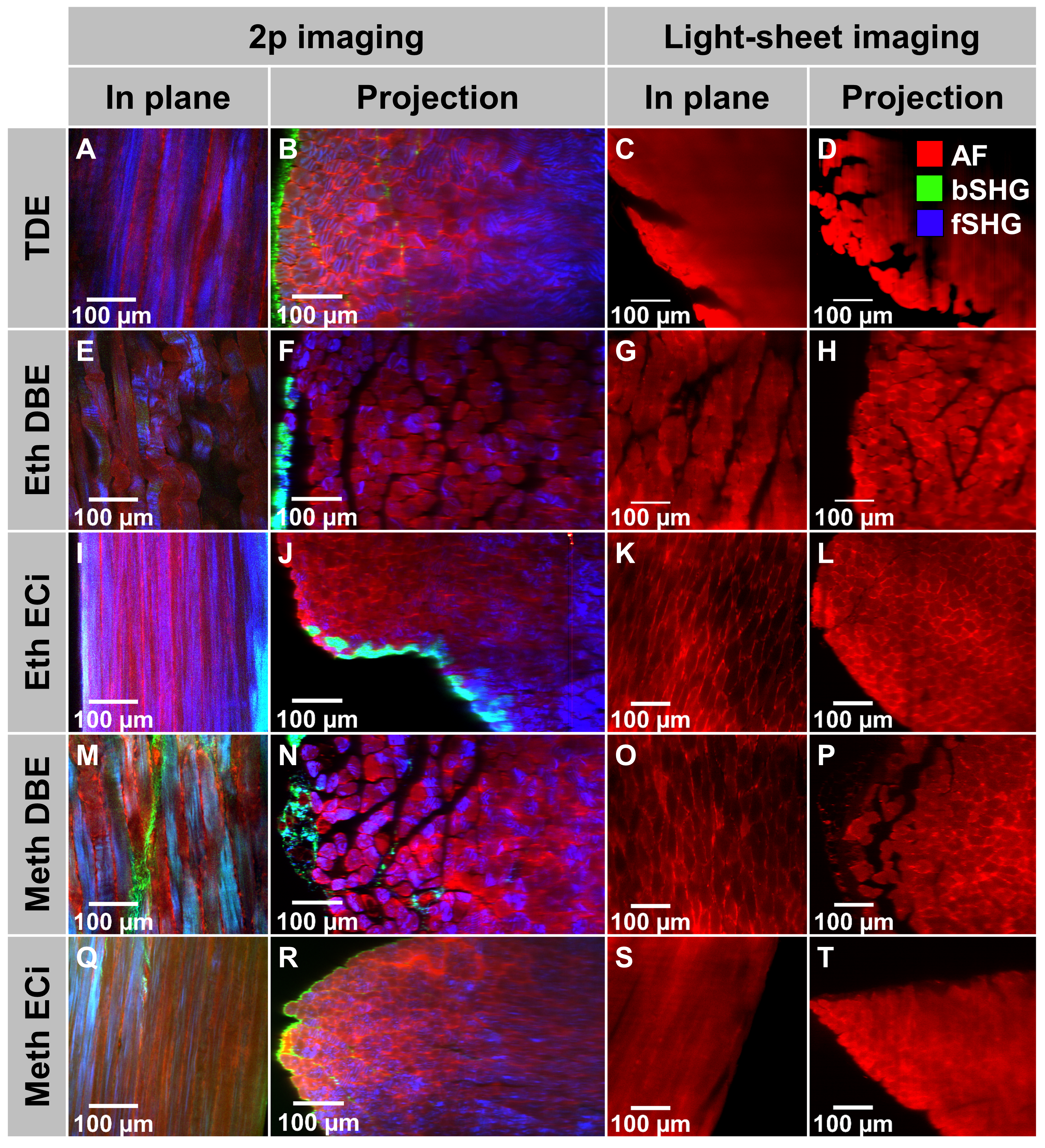

Supplement: Supplementary file 5 — Supplemental high-quality figure 5. [file thnov11p2876s5.png]
